# Advanced practice nursing in Latin America and the Caribbean: regulation, education and practice

**DOI:** 10.1590/1518-8345.1615.2807

**Published:** 2016-08-08

**Authors:** Keri Elizabeth Zug, Silvia Helena De Bortoli Cassiani, Joyce Pulcini, Alessandra Bassalobre Garcia, Francisca Aguirre-Boza, Jeongyoung Park

**Affiliations:** 1 Master's Student, School of Nursing, University of Pennsylvania, Philadelphia, PA, USA.; 2 Regional Advisor for Nursing and Allied Health Technicians, Pan American Health Organization, Washington DC, USA.; 3 Professor, School of Nursing, George Washington University, Washington DC, USA.; 4 Doctoral Student, Gillings School of Global Public Health, University of North Carolina, Chapel Hill, NC, USA.; 5 Professor, Universidad de los Andes, Santiago, Chile.; 6 Assistant Professor, School of Nursing, George Washington University, Washington DC, USA.

**Keywords:** Nursing, Public Health, Latin America, Caribbean Region, Advanced Practice Nursing, Community Health Nursing

## Abstract

**Objective:**

to identify the current state of advanced practice nursing regulation, education and practice in Latin America and the Caribbean and the perception of nursing leaders in the region toward an advanced practice nursing role in primary health care to support Universal Access to Health and Universal Health Coverage initiatives.

**Method:**

a descriptive cross-sectional design utilizing a web-based survey of 173 nursing leaders about their perceptions of the state of nursing practice and potential development of advanced practice nursing in their countries, including definition, work environment, regulation, education, nursing practice, nursing culture, and perceived receptiveness to an expanded role in primary health care.

**Result:**

the participants were largely familiar with the advanced practice nursing role, but most were unaware of or reported no current existing legislation for the advanced practice nursing role in their countries. Participants reported the need for increased faculty preparation and promotion of curricula reforms to emphasize primary health care programs to train advanced practice nurses. The vast majority of participants believed their countries' populations could benefit from an advanced practice nursing role in primary health care.

**Conclusion:**

strong legislative support and a solid educational framework are critical to the successful development of advanced practice nursing programs and practitioners to support Universal Access to Health and Universal Health Coverage initiatives.

## Introduction

Universal Access to Health and Universal Health Coverage (Universal Health) call for increased capacity of countries to provide high quality primary health care (PHC) while promoting health service delivery that is more accessible, equitable and efficient. Motivated and competent nurses can effectively deliver PHC to populations, supporting Universal Health initiatives worldwide. Based on this premise, the Pan American Health Organization (PAHO) released a Resolution in September 2013, Resolution CD52.R13: *Human Resources for Health: Increasing Access to Qualified Health Workers in Primary Heath Care Based Health Systems* ^1^, calling for an increased number of advanced practice nurses (APNs) to support PHC based systems.

PHC is defined by the World Health Organization (WHO) as "essential health care based on practical, scientifically sound and socially acceptable methods and technology made universally accessible to individuals and families in the community through their full participation and at a cost that the community and country can afford to maintain at every stage of their development in the spirit of self- reliance and self-determination. It forms an integral part both of the country's health system, of which it is the central function and main focus, and of the overall social and economic development of the community. It is the first level of contact of individuals, the family and community with the national health system bringing health care as close as possible to where people live and work, and constitutes the first element of a continuing health care process"^2^.

The International Council of Nurses (ICN) defines the advanced practice nurse as "a registered nurse who has acquired the expert knowledge base, complex decision-making skills and clinical competencies for expanded practice, the characteristics of which are shaped by the context and/or country in which s/he is credentialed to practice. A master's degree is recommended for entry level"^3^.

Each country uses different terminology to identify the role of the APN. One research study found 13 different titles for this role globally, including advanced nurse practitioner, nurse specialist, professional nurse, nurse practitioner (NP) to name a few^4^. In some countries, APNs are further subdivided into roles and specialties, such as hospital/acute care, mental health, pediatrics, midwifery/women's health, as well as PHC^4^. The APN in most countries has had training to support an expanded scope of practice beyond that of the nurse with a bachelor's degree. 

The aim of this exploratory, descriptive study was to identify the current state of APN regulation, education, and practice in Latin America and the Caribbean (LAC) and the potential for development of this role, particularly in the provision of PHC. To do so, the survey was conducted in 26 countries in the LAC region.

### Literature Review

Nurses have fulfilled a key role worldwide by providing PHC services in urban, rural, and underserved areas long before the existence of a formal APN role. In many countries, scope of practice was unregulated and nurses pursued the skills and expertise most pertinent to the needs of their population^5^. In recent decades, those seeking to formalize this role in some countries have urged hospitals, universities, and policymakers to support formally recognized APN programs for PHC^6^. Countries such as the United States of America and Canada have actively incorporated the APN role into their health care systems to provide PHC for their populations, with an emphasis on the most underserved communities. With over 50 years of experience incorporating APNs, the role in the United States was built upon the role of public health nurses. Currently, over 205,000 nurse practitioners (NPs) are licensed in the United States, two-thirds of whom practice in PHC^6^. Regulations for practice exist in all 50 states and the District of Columbia, and at least 21 states and the District of Columbia allow for full practice authority^7^. The United Kingdom, Canada and Australia also have systems to utilize APNs but progress in other parts of the world varies especially in use of APNs in primary health care^4^. Ample evidence-based research demonstrates that NPs predominantly work in PHC, provide high-quality, cost-effective care yielding comparable or better patient outcomes than their physician counterparts^5^. However, the APN roles in LAC countries have not yet been well established or well recognized. With the passage of PAHO's Resolution promoting more APNs to provide PHC, PAHO/WHO and other international organizations and partners have redoubled efforts to establish, promote, implement and recognize the APN role.

The NP role has been promoted with varying degrees of success in two Caribbean countries: Jamaica and Belize. Jamaica introduced the NP role in the 1970s due to physician shortages in rural and underserved areas. The two-year program offered by the University of the West Indies School of Nursing was converted to a master's level program in 2002^8^. However, NPs in Jamaica are still unable to legally prescribe medications without physician oversight and their effective integration into the health care system never fully came to fruition^9^.

The Belize School of Nursing first offered a Psychiatric Nurse Practitioner Certificate program in 1992, training 16 psychiatric NPs in collaboration with the Ministry of Health. The psychiatric NP's role is specifically tailored to address mental health needs of the population through outpatient consultation, and has effectively reduced the demand for inpatient psychiatric services in Belize^10^.Psychiatric NPs do possess prescriptive authority, but only for psychotropic medications^11^. However, the psychiatric NP certification is earned via a certificate program; a master's degree is not required for practice. Role confusion with that of psychiatric nurses and comparably low financial compensation for psychiatric NPs have not incentivized prospective applicants to the program in Belize^10^. Standardized education, role clarification and wage reform are integral to permanently establishing this and other expanded nursing roles.

Regarding APNs in public health, it is important to note that the field of public health varies across the region, according to local, regional and national demands of the health system and is influenced by a broad range of cultural, historic and economic climates^12^. In many LAC countries, the public health nursing role is multifaceted, including disease prevention, patient education, managing immunization programs (including vaccination administration), and in some cases, home visitation^12^ ^-^ ^13^. However, public health nurses have limited recognized professional autonomy, which restricts their ability to diagnose, construct management plans and prescribe medications^13^. Public health nurses are the largest group of professionals in public health within the region and express frustration at the lack of a clear public health nurse job description, which seems to vary depending on the health system infrastructure region to region^13^.

Many countries in the LAC region have established higher education nursing degrees. Nursing programs at the master's level have existed in the region since the 1972, and doctoral-level programs were introduced in the eighties with the University of São Paulo in Brazil initiating the first doctorate-level nursing program in 1982^14^. Doctorate level nursing degrees have since been introduced in Argentina, Colombia, Cuba, Chile, Mexico, Peru and Venezuela^14^.

Although graduate programs in LAC might not be actively changing the scope of nursing practice at the regulation level, they are still promoting professional development, research, leadership and improving clinical decision-making at the practice level^14^ ^-^ ^15^. Nurses with graduate degrees often enter managerial roles, or become professors or researchers at universities^15^. Several nations in LAC have specialization certificate programs for nursing specialty areas, but most of them are for hospital-based specialties as opposed to primary care and do not appear to expand scope of practice in PHC^15^.

In April 2015, PAHO/WHO, the Canadian Government, and McMaster University collaborated to promote discussion among nursing leaders from LAC at the Universal Access to Health and Universal Health Coverage Advanced Practice Nursing Summit in Hamilton, Canada. Strategies were established to best introduce and integrate the APN role in LAC to fulfill PAHO's 2013 Resolution^1^. Since the conference, a framework for collecting further data and planning implementation of the APN role in LAC has been established and future steps toward ongoing collaborations pursued^16^. Several countries such as Brazil, Mexico, Colombia and Chile have begun their own discussions to explore the viability of introducing the APN role in their national model of health care^17^.

## Methods

This study used a descriptive cross-sectional design using a web-based survey via SurveyMonkey about the state of the APN and registered nurse in LAC. The focus of the survey was role definition, work environment, regulation, education, nursing practice, nursing culture, and perceived receptiveness to an expanded role in PHC.

In addition to the definition of PHC, the survey introduction included the ICN definition of the APN to guide participants in developing a common understanding of this role. The survey contained 26 objective questions and 3 qualitative questions. The participants were not required to answer all of the questions in order to complete the survey. The qualitative data will be presented in a future paper. The survey was pilot tested in English for understandability of language and terminology by 6 nurses prior to finalization of survey wording. After recommendations from the pilot surveys were considered and implemented, the survey was translated from English to Portuguese and Spanish. Master's level-educated nurses from LAC performed the translation. The two translators are well versed in health care terminology, and fluent both in English and in their native language (Portuguese and Spanish, respectively).

### Participants/Sample

**Figure 1:**
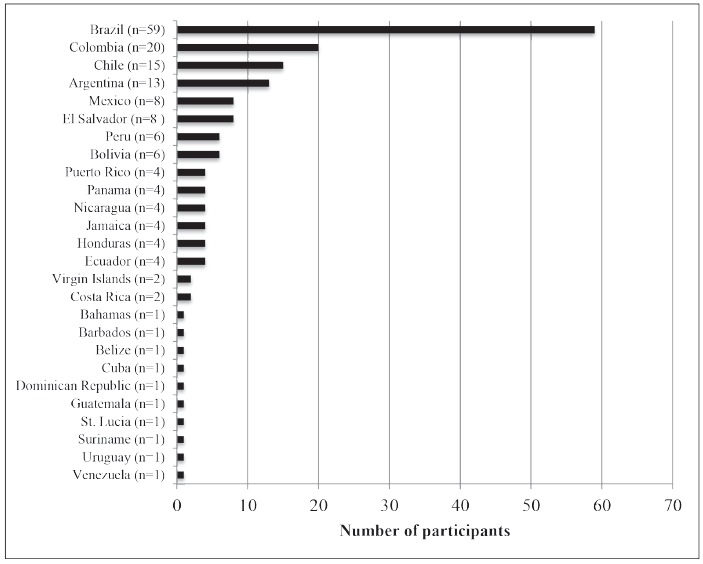
Number of participants by country, 2015

Using convenience sampling with a snowball technique, the initial contacts were asked to send the survey on to five more nursing leaders or key informants in their country to extend the reach of the survey to influential voices in nursing leadership identified by the primary contacts. The initial sample was drawn from the PAHO/WHO Nursing Network list. A final sample of 173 persons from 26 countries was obtained after distributing the survey in English, Spanish, and Portuguese to 468 nursing leaders in LAC (response rate: 37%). Figure 1 depicts the number of participants by country.

The majority of participants were highly educated with 43% (n=75) possessing doctorate or post-doctorate degrees and another third posessing other graduate level degrees. Of the 16% (n=28) who responded "other" to this survey question, most indicated they were doctoral students, nurses with specializations or nurses with other types of certification. 

**Figure 2:**
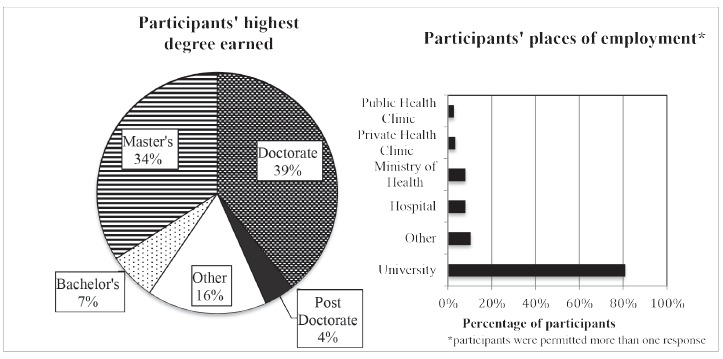
Education level and place of employment of participants

The vast majority of participants (81%, n=140) indicated they were university employed and 70% (n=121) indicated they worked as educators. Eight percent (n=14) worked in Ministries of Health and 7% (n=12) worked as policymakers. Participants who selected "other" indicated they were heads and officers of nursing associations as well as key players in regional and local health initiatives. Figure 2 depicts education level and place of employment of participants.

### Human Subjects

Participants were asked to consent to the survey after being notified of the study procedures, confidentiality protections and potential risks and benefits of the survey. No identifying information was collected about the subject except for demographics and country of origin. The survey received an exempt review by the George Washington University Institutional Review Board and by PAHO/WHO prior to being administered.

### Data Analysis

A descriptive analysis of current regulation/legislation, roles, education, perception, and barriers and facilitators to APN roles using a 2015 survey of nursing leaders in LAC was undertaken. Because the study focused on describing the current state of APN in LAC, statistical tests or report confidence intervals were not performed. Since the vast majority of participants (62%) came from Brazil, Colombia, Chile, and Argentina, an analysis after excluding these 4 countries was also conducted. Overall, the results are not substantially different for the vast majority of the survey questions. Thus, the main results are presented based on the data from all 26 participating countries. Where the responses did differ, however, the data are presented from countries after excluding Brazil, Colombia, Chile and Argentina in addition to the data from all countries. Data were analyzed using Stata 13.

## Results

### Regulation/Legislation

Participants were questioned about current regulation and legislation for licensed nursing (bachelor-educated nursing) roles as well as the existence or development of legislation for APN roles. The majority of participants (88%, n=143) indicated regulation and regulatory bodies existed for licensed nursing practice in their countries, yet there was no consensus in terms of the participants' perceptions of existing or planned APN regulation. While the majority of participants were familiar with the APN Role (88%, n=151), over half of participants (51%, n=80) indicated no legislation currently existed to regulate the APN role, while 25% (n=39) were unsure if present legislation addresses APNs. Twelve percent (n=19) of participants indicated legislation existed and 11% replied it was currently in development in their country (n=18).

### Regulation and Nursing Roles

Participants were asked about title protection of the licensed nursing role and whether role distinction for the licensed nurse and the nurse auxiliary were explicitly delineated in the regulatory acts and in practice settings. The presence of clear role and responsibility distinction for current nursing and nurse auxiliaries bodes well for the future establishment of and regard for discernable areas of role expansion for the APN. Seventy-five percent of participants agreed that title protection did exist in their countries (n=114). Their response differed in terms of role distinction in the care delivery setting, particularly in Brazil where although 89% (n=48) of participants believed legislation existed to delineate clear role distinction, only 57% (n=32) reported that role distinction was clear in the practice setting. Among all participants, only 73% (n=115) reported that legislation addressed clear role distinction between licensed nurses and nurse auxiliaries, while only 58% (n=94) reported it existed in health care delivery environments.

### Primary Health Care Basis in the Nursing Education

**Figure 3:**
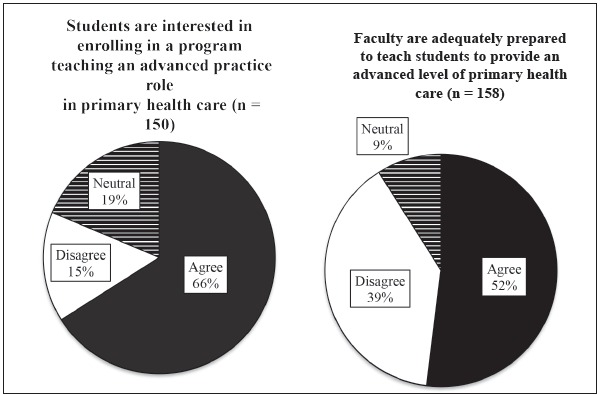
Primary health care education: Student interest and faculty preparedness

The survey asked participants about their perceptions of Bachelor's of Science in Nursing (BSN) education in their country addressing PHC. The vast majority of participants (93%, n=147) indicated that BSN programs in their countries required students to have a PHC or community health clinical rotation. Participants were also asked about student interest in enrolling in a program teaching an advanced level of PHC and as to the preparedness level of the faculty in teaching this material. The majority of participants agreed students are interested in an advanced nursing degree to provide PHC, but many participants indicated they were not confident in the faculty's capacity to teach at this level, as depicted in Figure 3. When this data was analyzed for all countries besides Brazil, Argentina, Chile and Colombia, 70% (n=43) reported that faculty were prepared to teach an advanced level of primary health care, while 26% disagreed (n=16). 

### Perception of the APN Role

**Figure 4:**
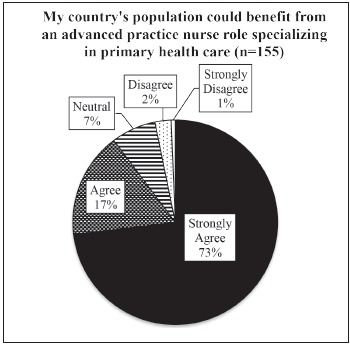
Perceptions of participants towards the benefits of APN role in their country

Participants were asked if they felt their country's populations could benefit from the introduction and implementation of the APN role. Overall, participants reported that the APN would be a beneficial addition to their health system and country populations, as illustrated in Figure 4.

### Barriers and Facilitators to the implementation of the APN Role

**Figure 5:**
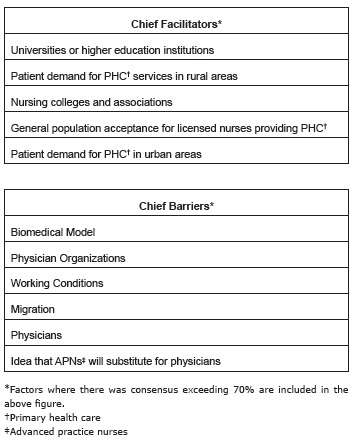
Chief facilitators and barriers for implementation of the advanced practice nursing role in surveyed countries as indicated by participants. Latin America and the Caribbean, 2015

The participants were given a list of factors potentially influencing the realization of the APN role in LAC and asked to indicate which would be considered top facilitators and barriers in its implementation. Figure 5 depicts their responses. In terms of facilitators, over 90% (n=156) felt universities or higher education institutions would be a driving force in supporting the implementation of the APN role. Also notable is the perception that patient demand for PHC in rural areas and urban areas as well as the general population's acceptance for licensed nursing providing PHC were considered top facilitators.

While there was somewhat less consensus on the barriers to APN implementation the chief factors indicated by participants were the biomedical model and the physician role. Current working conditions and migration were also indicated as top barriers (Figure 5).

## Discussion

APNs can deliver PHC, making quality healthcare more accessible, equitable and efficient. PAHO/WHO Resolution CD52.R13 calls for increasing the number of APNs to support PHC based systems. In LAC, this role has not been well established or recognized, which creates a major challenge for the region. More than half of participants stated that there is no legislation to regulate the role of APN, and another quarter indicated they were not aware of any legislation addressing this role. Legal protection through the establishment of regulation governing the APN role is essential to formalizing the role in LAC countries.

Moreover, it is interesting that only 58% (n=94) of respondents feel that their role is clear in the practice setting. In order to implement and recognize the role of APN, the role of the licensed nurse should first be clarified, especially in the PHC setting. Clarification of the role of the PHC nurse in each country could be a next step and would be an influential area for future research.

Another problem revealed by the survey is that many participants were not confident in the faculty's capacity to teach an advanced level of primary health care; especially given that this sample is composed of over 80% of participants coming from the university setting. To successfully implement the APN role in LAC, competent master's or doctoral level-prepared teachers are needed to prepare future APNs. It is evident that LAC countries may need to seek help from foreign universities that may assist with generating first cohorts of teaching faculty and graduates. Medical schools in LAC could also be recruited to assist with this task, as occurred in the United States when the first APN programs were developed^18^. Working with physician colleagues or associations from the beginning can be essential in accomplishing the task of implementing the APN role.

The barriers and facilitators expected by participants in the implementation of the APN role do not prove to be different than those cited in the global literature ^4^ ^,^ ^12^. Barriers such as the biomedical model, the role of physicians, working conditions and migration are common themes. Knowing experiences of countries that have already implemented the APN role puts LAC countries in an advantageous position to work proactively to overcome the identified barriers, and capitalize on the facilitators.

The development of the APN in LAC need not replicate what took place in Canada or the United States, but can instead build upon lessons learned from these countries' experiences. It is imperative to encourage collaborative partnerships between nursing associations, universities and the Ministries of Health both regionally, nationally and internationally in the establishment of the APN role to meet primary health care priorities for individual countries in LAC.

### Limitations

Our survey was limited by some factors which may partially compromise its external validity. We utilized a combined method of convenience sampling using a snowball technique to reach key informants as well as other nursing leaders identified by our key informants. In addition, a larger response was received from participants in countries with a larger nursing presence, hence more nursing leaders and key informants were contacted from four countries: Brazil, Colombia, Argentina, Chile. This did lead to data skewing toward some countries when results from participants are presented (particularly Brazil), but we also examined the data when these four countries were excluded and found the results to not be substantially different. The survey did not have participants from all countries in the LAC region, so the results may not be truly representative of the entire region, although useful for a general representation.

The participants were largely employed by universities, and thus did not represent a true cross section of nursing leaders from different disciplines, such as practice, policy and administration that may have provided a more nuanced perspective. Even though the response rate was not high, it was adequate for the purpose of informing educators and policymakers.

## Conclusion

This is the first comprehensive international survey of LAC nursing leaders' perceptions of a potential APN role to provide PHC to their populations. Critical to the successful development of APN programs and practitioners are strong legislative support and a solid educational framework that must continue to inform one another. 

Overall, participants indicated nursing regulatory bodies do exist, although role distinction challenges in the workplace persist. In regard to an APN role for PHC, participants report a lack of planned legislation for an expanded APN scope, but the vast majority felt their country's populations would benefit from this role. In terms of education, the survey indicated participants feel programs are adequately emphasizing PHC and that students would be interested in this type of APN role.

Considering most of our participants were affiliated with universities or were nursing leaders in their countries, this bodes well for receptivity to initial conversations about planning for an APN education at the graduate level. Areas for further consideration include curricula review and faculty preparedness to teach an advanced level of PHC.

The term "advanced practice nursing" is not well recognized in LAC countries and the APN is a relatively new role in the region. PAHO/WHO is working with the individual LAC countries to learn from the experiences and research that Canada and USA have provided on this topic and working with nursing associations, nursing educators and leaders from the Ministries of Health and Education in the various countries.

It will be a long journey for the role of the APN in LAC countries to become established, implemented and well positioned in the health care system, but the development of this role is a significant step toward achieving Universal Health in the region.
